# Real-World Outcomes of First-Line Pembrolizumab Monotherapy in Metastatic NSCLC with High PD-L1 Expression (TPS ≥ 50%): A Multicenter Study from Serbia

**DOI:** 10.3390/biomedicines13051175

**Published:** 2025-05-11

**Authors:** Filip Marković, Mihailo Stjepanović, Milan Rančić, Marina Cekić, Milica Kontić

**Affiliations:** 1 Clinic for Pulmonology, University Clinical Centre of Serbia, 11000 Belgrade, Serbia; mihailostjepanovic@gmail.com (M.S.); milicakontic@yahoo.com (M.K.); 2 Faculty of Medicine, University of Belgrade, 11000 Belgrade, Serbia; 3 Pulmonary Diseases Clinic, University Clinical Center Nis, 18000 Nis, Serbia; milanrancic@gmail.com (M.R.); marina.cekic@gmail.com (M.C.); 4 Faculty of Medicine, University of Nis, 18000 Nis, Serbia

**Keywords:** first-line, high PD-L1 expression, immune check-point inhibitors, non-small cell lung cancer, pembrolizumab, predictive factors

## Abstract

**Background:** Pembrolizumab monotherapy is the standard first-line treatment for metastatic non-small cell lung cancer (NSCLC) patients whose tumors express a PD-L1 tumor proportion score (TPS) of ≥50%. However, real-world data regarding its effectiveness outside of clinical trials, particularly in Eastern European populations, are limited. **Methods:** We conducted a retrospective, multicenter study including 225 patients with metastatic NSCLC and PD-L1 TPS ≥ 50% who received first-line pembrolizumab monotherapy in Serbia between 2019 and 2022. Patient demographics, clinical characteristics, and treatment outcomes were collected. Progression-free survival (PFS) and overall survival (OS) were estimated using the Kaplan–Meier method, and multivariable Cox proportional hazards regression was performed to identify predictors of outcomes. **Results:** The median PFS was 9.7 months (95% CI: 7.979–11.421), and the median OS was 17.0 months (95% CI: 12.813–20.187) at a median follow-up of 18.1 months. The overall response rate (ORR) was 36.4%, and the disease control rate (DCR) was 73.4%. Multivariable analysis identified good performance status (ECOG PS 0–1), PD-L1 TPS ≥ 90%, and the occurrence of immune-related adverse events (irAEs) as independent predictors of improved PFS and OS. **Conclusions:** Our study highlights the efficacy and safety of first-line pembrolizumab monotherapy in a real-world Serbian population with metastatic NSCLC and high PD-L1 expression. Furthermore, it confirms the prognostic value of ECOG PS, high PD-L1 expression, and the development of irAEs in predicting favorable clinical outcomes.

## Simple Summary

This study analyzed real-world outcomes in 225 patients with metastatic non-small cell lung cancer (NSCLC) and a high PD-L1 tumor proportion score (TPS ≥ 50%) who were treated with first-line pembrolizumab monotherapy between 2019 and 2022 in Serbia. The median progression-free survival (PFS) was 9.7 months, and the median overall survival (OS) was 17.0 months. The overall response rate (ORR) was 36.4%, and the disease control rate (DCR) was 73.4%. Patients with good performance status (ECOG PS 0–1), PD-L1 TPS ≥ 90%, or those who developed immune-related adverse events (irAEs) had significantly better outcomes. These findings confirm the effectiveness and safety of pembrolizumab in routine clinical practice and highlight clinical factors associated with improved survival in this population.

## 1. Introduction

Lung cancer remains a major global health challenge, as it is the most frequently diagnosed cancer and the leading cause of cancer-related mortality worldwide [1]. Despite advances in screening and therapeutic strategies, the prognosis for lung cancer patients remains poor, particularly among those diagnosed at advanced stages [1]. Non-small cell lung cancer (NSCLC) accounts for approximately 80–85% of all lung cancer cases, with the majority of patients presenting with advanced or metastatic disease at the time of diagnosis [2,3]. Historically, systemic chemotherapy has represented the mainstay of treatment, offering limited survival benefits and considerable toxicity.

The advent of immune checkpoint inhibitors (ICIs) has profoundly transformed the treatment landscape for advanced NSCLC [4]. By targeting key regulatory pathways involved in immune evasion, such as the programmed death-1/programmed death-ligand 1 (PD-1/PD-L1) axis, ICIs have led to unprecedented improvements in survival for certain subsets of patients. Pembrolizumab, a humanized monoclonal antibody against PD-1, became the first ICI approved as a first-line monotherapy for patients with high PD-L1 expression (tumor proportion score [TPS] ≥ 50%) and no actionable driver mutations, based on the results of the KEYNOTE-024 trial [5]. In this pivotal randomized controlled trial, pembrolizumab demonstrated superior progression-free survival (PFS) and overall survival (OS) compared to platinum-based chemotherapy, establishing a new standard of care [5]. Long-term follow-up from the KEYNOTE-024 reported a median OS of 26.3 months and a 5-year survival rate of 31.9%, highlighting the potential for durable benefit in selected patients [6].

While these results marked a significant milestone, the highly selected patient populations enrolled in randomized controlled trials (RCTs) do not fully reflect the heterogeneity seen in routine clinical practice. Patients with poorer performance status, significant comorbidities, or other adverse prognostic factors are often underrepresented in clinical trials [7]. Consequently, real-world studies have emerged as an essential complement to RCTs, providing critical insights into the effectiveness and safety of pembrolizumab in broader, unselected patient populations [7,8,9]. These real-world investigations have generally confirmed the clinical benefit of pembrolizumab monotherapy; however, outcomes are often less favorable than those reported in RCTs, underscoring the influence of patient- and system-related factors on treatment success [7,8,9]. Despite the growing body of real-world data globally, there remains a notable lack of evidence from Eastern Europe, including Serbia, which has one of the highest lung cancer incidence rates worldwide [3]. However, the availability of real-world data on immunotherapy outcomes in this region is scarce. Differences in healthcare infrastructure, patient demographics, disease characteristics, and access to innovative therapies may influence outcomes and thus warrant specific investigation.

To address this knowledge gap, we conducted a multicenter retrospective study evaluating the real-world outcomes of first-line pembrolizumab monotherapy in patients with metastatic, non-oncogene-addicted NSCLC and PD-L1 TPS ≥ 50% treated at the two largest lung cancer centers in central and southern Serbia. Our objectives were to assess patient characteristics, treatment efficacy, and the safety profile of pembrolizumab in routine clinical practice, as well as to identify clinical factors associated with improved survival in this specific population.

## 2. Materials and Methods

The study included patients with histologically confirmed metastatic NSCLC with PD-L1 TPS ≥ 50% that started treatment with pembrolizumab monotherapy between 2019 and 2022. The study was conducted at two academic institutions in Serbia. Data were retrieved from hospital-based lung cancer registries.

All patients underwent routine PD-L1 testing performed on formalin-fixed, paraffin-embedded histology or cytology samples using PD-L1 monoclonal antibodies (22C3 clone by DAKO, Glostrup, Denmark) prior to initiating the first line of treatment. Patients were tested for EGFR mutations by Cobas^®^ EGFR Mutation Test v2 and ALK rearrangements by immunohistochemistry prior to first-line treatment initiation.

Patients with detected driver oncogenes were excluded, so none of the included patients had a known driver oncogene. All patients were followed and assessed for best response to therapy according to RECIST v1.1 criteria as per local practice [1]. Toxicity was graded according to the Common Terminology Criteria for Adverse Events (CTCAE) v5.0 [2].

### 2.1. Ethics Approval

Data on patients with newly diagnosed lung cancer are prospectively collected within a clinical lung cancer registry that collects demographics, pathological and molecular characteristics, as well as treatment and survival data for all patients with lung cancer diagnosed and treated at the center. All data were collected anonymously. The study was performed in accordance with the Declaration of Helsinki and approved by the Institutional Review Board (2268/2; 26 July 2024).

### 2.2. Statistical Analysis

Descriptive methods were used to analyze demographic characteristics of patients. Baseline information is presented as the number of patients and percentages. Median progression-free (PFS) and overall survival (OS) were calculated as the time from the start of therapy until disease progression or death. Patients still alive on the last day of follow-up were censored. Median PFS and OS were estimated by the Kaplan–Meier method and compared using the log-rank test. Univariable and multivariable Cox proportional hazard regression models were used to calculate hazard ratios (HRs) and confidence intervals (CIs). In the univariate analysis, covariates included age (<70 years vs. ≥70 years), sex, histology (non-squamous vs. squamous cell lung cancer), smoking status (current or former vs. never-smoker), PD-L1 expression (TPS 50–90% vs. ≥90%), ECOG PS (0–1 vs. ≥2), the presence of brain metastasis at baseline (yes vs. no), radiotherapy during treatment (yes vs. no), corticosteroid use at the time of treatment (yes vs. no), and the occurrence of immune-related adverse events (yes vs. no). Multivariate analysis included variables with a significance level of *p* < 0.10 in the univariate analysis. The chi-square test was used to determine the association between response to treatment and patient age. Calculated *p*-values were two-sided. We used SPSS v26 for statistical analysis.

## 3. Results

There were 225 patients with metastatic NSCLC and PD-L1 TPS ≥ 50% without detectable oncogene addiction who started treatment with pembrolizumab monotherapy as first-line treatment. The mean age was 64.77 (range 41–90) years, and 64% of patients were male. Most of them were either current or former smokers, 59.1% and 25.7%, respectively. Regarding histology, adenocarcinomas were detected in 61.7% and squamous cell carcinomas in 30.6% of cases. There were 42 (18.67%) patients with poor performance status, defined as Eastern Cooperative Oncology Group (ECOG) performance status (PS) ≥ 2, and corticosteroid use at the time of start of therapy was noted in 35 (15.5%) patients (Table 1).

Comparison of baseline characteristics between patients with and without immune-related adverse events (irAEs) (Table 2) showed no statistically significant differences regarding sex, smoking status, age, histology, number of metastatic sites, PD-L1 TPS, ECOG performance status (PS), radiotherapy use, or corticosteroid use at therapy initiation. Similarly, when comparing patients according to ECOG PS (Table 3), patients with ECOG PS ≥ 2 were significantly older (*p* = 0.01) and more frequently required corticosteroid use at treatment start (40.4% vs. 9.8%, *p* < 0.001). No significant differences were observed between the groups in terms of sex, smoking history, histology, number of metastatic sites, irAE occurrence, PD-L1 TPS, or radiotherapy use. Finally, in the comparison of patients with PD-L1 TPS ≥ 90% versus those with TPS 50–89% (Table 4), no statistically significant differences were identified across baseline characteristics, including corticosteroid use. Overall, poorer PS was associated with older age and higher baseline corticosteroid use, while occurrence of irAEs and PD-L1 TPS levels did not show significant associations with other clinical or demographic factors.

**Figure 1:**
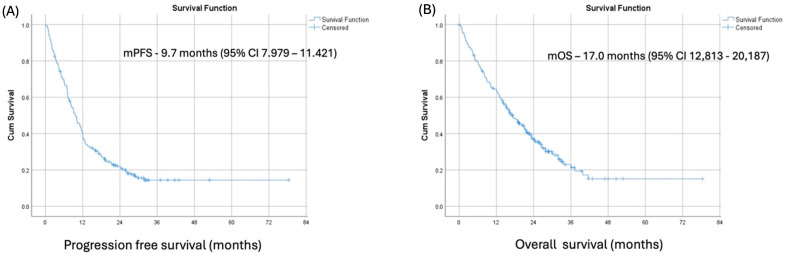
Kaplan–Meier survival curves for progression-free (**A**) and overall survival (**B**) for the whole cohort.

At a median follow-up of 18.1 months (range from 0.1 to 71.2 months), the median PFS and median OS in our patient cohort were 9.7 months (95% CI 7.979–11.421) and 17.00 months (95% CI 12,813–20,187), respectively. (Figure 1) The best overall responses to pembrolizumab were complete response, partial response, and stable disease in 3.1%, 33.3%, and 37%, respectively, resulting in an overall response rate (ORR) of 36.4% and disease control rate (DCR) of 73.4%.

A total of 61 immune-related adverse events (irAE) were observed in 47 patients (20,8%), with 13 patients experiencing more than one irAE. The most common irAEs were skin toxicities (17/47 patients; 36,1%), thyroid dysfunction (9/47 patients; 19,1%), colitis (9/47 patients; 19,1%), and hepatotoxicity (8/47 patients;17%). Most of the recorded irAEs were of grade 1–2 (55/61; 90.1%) as per CTCAE v5 [2] (Table 5). Pembrolizumab therapy was permanently discontinued in 3 cases of grade 3–4 hepatotoxicity and 1 case of grade 3–4 pneumonitis as per ESMO Guidelines for the management of toxicities from immunotherapy [3].

**Figure 2:**
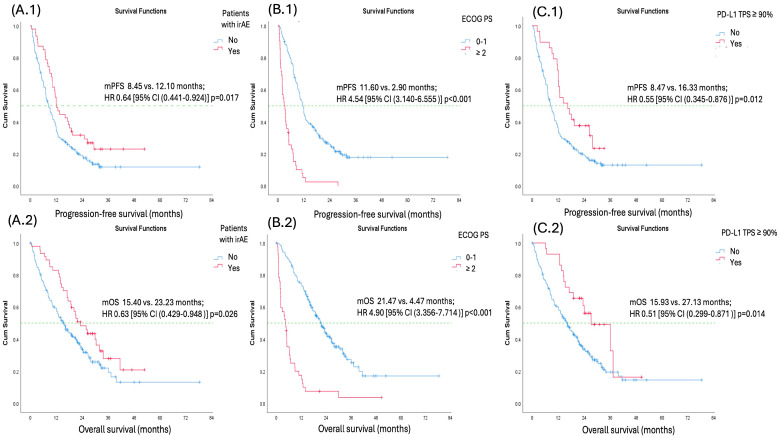
Kaplan–Meier survival curves showing progression-free (**A.1**) and overall survival (**A.2**) for patients who experienced irAEs compared to those who did not, progression-free (**B.1**) and overall survival (**B.2**) for patients with ECOG 0–1 versus ECOG ≥ 2, and progression-free (**C.1**) and overall survival (**C.2**) for patients with PD-L1 TPS 50–89% versus ≥90%.

Univariate analysis for both PFS and OS identified the occurrence of irAEs, good performance status (ECOG PS 0–1), PD-L1 TPS ≥ 90%, and corticosteroid use at the time of therapy initiation as factors associated with favorable PFS and OS. However, in multivariate analysis for both PFS and OS, only the occurrence of irAEs, good performance status (ECOG PS 0–1), and PD-L1 TPS ≥ 90% remained significant predictors. Factors such as sex, histology, smoking status (ever vs. never-smokers), number of metastatic sites (<3 vs. ≥3 sites), corticosteroid use at the time of therapy initiation, radiotherapy, and presence of CNS metastases were not predictive of better PFS or OS (Table 6 and Table 7) (Figure 2).

## 4. Discussion

In our study, patients with metastatic NSCLC and PD-L1 TPS ≥ 50% treated with first-line pembrolizumab monotherapy had a median PFS of 9.7 months (95% CI: 7.979–11.421) and a median OS of 17.0 months (95% CI: 12.813–20.187), both of which were lower than those reported in the pivotal KEYNOTE-024 trial [4,5]. Multiple real-world registry studies assessing this treatment in the same patient population have also reported lower median PFS and OS compared to the pivotal KEYNOTE-024 trial [6,7,8,9,10,11]. This disparity may be attributed to the inclusion of patients with characteristics such as poor ECOG performance status, who were excluded from the pivotal randomized clinical trial [4,5]. Our findings regarding median PFS and median OS are comparable with those recently reported by authors of a large multicenter Spanish nation-wide study that included 494 advanced NSCLC patients with PD-L1 TPS ≥ 50% that underwent pembrolizumab monotherapy in the first line of treatment [12].

Reports indicate that patients with poor ECOG PS (≥2) make up to 30% of those diagnosed with advanced NSCLC, yet they remain underrepresented in large-scale clinical trials of first-line ICIs [4,12]. Despite this, real-world data consistently show that ECOG PS ≥ 2 is associated with poorer treatment outcomes in advanced NSCLC patients receiving ICI therapy, which aligns with our findings [6,7,10,12,13,14,15,16,17].

Poor ECOG performance status likely reflects a greater burden of NSCLC, a higher prevalence of comorbidities, diminished physiologic reserve, and a reduced capacity to recover from disease progression or treatment-related complications. Although we did not assess hospitalization rates or emergency department visits in our cohort, prior studies suggest that advanced NSCLC patients undergoing ICI therapy with an ECOG PS ≥ 2 are significantly more likely to experience adverse clinical outcomes following ICI initiation, including increased rates of ED visits, hospital admissions, and in-hospital mortality compared to those with ECOG PS 0–1 [18,19].

In our cohort, older age (≥70 years) and higher corticosteroid use at baseline were associated with ECOG PS ≥ 2. This may be because both factors are related to greater baseline frailty and disease burden. Older patients often have more comorbidities (like chronic pulmonary disease, cardiovascular disease, or arthritis) that can impair daily functioning, thus leading to a worse ECOG PS. Similarly, corticosteroids are frequently prescribed to manage cancer-related symptoms (e.g., dyspnea, fatigue, appetite loss, brain metastasis-related edema) or to control other comorbid conditions, all of which are more common or severe in patients who already have a poor functional status.

A similar association between ECOG PS ≥ 2 and baseline corticosteroid use was reported by De Giglio et al., who found that corticosteroid use at baseline had a detrimental effect on both median progression-free survival (mPFS) and median overall survival (mOS) [20]. In contrast, we did not observe such an association in our cohort, which may be explained by the lower incidence of baseline corticosteroid use in our study (15.6%) compared to that reported by De Giglio et al. (35.6%) [20].

The phase 3 IPSOS trial provided valuable insights into treatment options for patients ineligible for platinum-based chemotherapy due to poor ECOG PS (≥2) as well as advanced age or significant comorbidities [17,21]. In this study, patients were randomized to receive either single-agent chemotherapy or first-line atezolizumab monotherapy, regardless of PD-L1 TPS. The results showed that first-line ICI monotherapy improved median OS (10.3 vs. 9.2 months; *p* = 0.028). These findings highlight that while ECOG PS ≥ 2 remains a poor prognostic factor, first-line ICI monotherapy may still be a viable option for select patients with advanced NSCLC who cannot tolerate platinum-based treatment.

The exact pathophysiology of irAEs is not fully elucidated. However, they are believed to be closely associated with the essential function of immune checkpoints in maintaining immunologic homeostasis [22]. The development of irAEs following treatment with ICIs is hypothesized to reflect a broadly activated immune response that may concurrently enhance antitumor activity. Consequently, their role as potential predictive biomarkers of ICI treatment outcomes has been the subject of extensive investigation among patients with neoplasms, including but not limited to advanced NSCLC [23,24,25,26,27,28].

Thyroid dysfunction rarely requires immunosuppressants because it is typically self-limiting and can often be effectively managed with thyroid hormone replacement. Similarly, dermatologic reactions are rarely severe and typically respond to local or short-term corticosteroid treatments without requiring the extended use of corticosteroids.

A retrospective multicenter study by Wang et al. and a meta-analysis by Zhang et al. found that patients experiencing low-grade irAEs derive the greatest clinical benefit from ICI monotherapy [26,28]. The majority of patients in our study experienced low-grade irAEs. This may explain the observed association of irAEs and improved outcomes in terms of mPFS and mOS. High-grade irAEs can be life-threatening and may necessitate hospitalization, ICI treatment discontinuation, and systemic immunosuppression, which may counteract the efficacy of ICIs and result in poorer clinical outcomes. Additionally, in a subgroup analysis, Zhang et al. reported that skin, endocrine, and gastrointestinal irAEs were associated with improved survival outcomes. A more recent meta-analysis by Lin et al., which also included ICI-based combination therapy for advanced NSCLC, reinforced the association between these types of irAEs and favorable outcomes [29]. Among our patients, skin, endocrine, and gastrointestinal irAEs were the most prevalent. Dermatologic irAEs are rarely severe and typically respond to local or short-term corticosteroid treatments without requiring the extended use of corticosteroids [3]. Thyroid-related irAEs are the most common among endocrine irAes. They rarely require immunosuppressants because they are typically self-limiting and can often be effectively managed with thyroid hormone replacement [3]. The most irAE affecting the gastrointestinal system is ICI-related colitis [30]. When diagnosed as a low-grade irAE, as was the case in most patients that experienced this irAE in our cohort, it can generally be managed with a low-fiber diet, loperamide, and short-term corticosteroid treatment, without requiring permanent discontinuation of ICI therapy [3]. The fact that thyroid, skin, and gastrointestinal irAEs typically do not necessitate prolonged immunosuppressive treatment and ICI discontinuation may play a key role in preserving the efficacy of ICIs.

A large-scale retrospective multicenter study by Cortellini et al., which analyzed 1010 advanced NSCLC patients with PD-L1 TPS ≥ 50% treated with first-line pembrolizumab monotherapy, demonstrated that irAEs were linked to improved median PFS and OS, which is also in line with our findings [27]. Moreover, the authors also found that the occurrence of multiple irAEs in a single patient was associated with improved outcomes compared to patients who experienced only one irAE. While there were 13 patients who experienced multiple irAEs in our cohort of patients, the sample was too small to carry out such an analysis and draw conclusions from it.

PD-L1 TPS guides first-line treatment decisions for NSCLC patients without targetable genomic alterations [31,32]. Despite this, PD-L1 TPS remains an imperfect predictor of treatment response and prognosis, largely due to intra- and intertumoral heterogeneity [33]. Notably, there is no consensus on the cutoff used to define very high PD-L1 TPS among advanced NSCLC patients without oncogene addiction and PD-L1 TPS ≥ 50%. Different studies applied varying thresholds, which may contribute to these conflicting findings in studies comparing outcomes among these patients based on PD-L1 TPS [10,16,34,35,36,37]. Nevertheless, consistent with our findings, several studies have reported an association between PD-L1 TPS ≥ 90% and improved outcomes in metastatic NSCLC patients receiving first-line pembrolizumab monotherapy compared to those with PD-L1 TPS 50–89% in terms of mPFS and mOS [38,39,40]. Shah et al. have recommended implementing PD-L1 stratified randomization (PD-L1 TPS 50–89% vs. ≥90%) and reporting outcomes accordingly in ongoing and future randomized controlled trials evaluating ICI-based regimens in this patient population to further evaluate predictive and prognostic capabilities of PD-L1 TPS ≥ 90% [40]. In a pooled analysis of 11 randomized controlled trials involving patients with advanced NSCLC receiving ICI monotherapy, Vallejo et al. reported that higher levels of PD-L1 expression were associated with improved PFS and OS [36]. The study also highlighted regional differences in the impact of PD-L1 expression on outcomes, underscoring the importance of conducting future trials in multiregional settings with diverse patient populations. In this context, it is equally important to report real-world data from regions underrepresented in clinical trials, as such data can provide critical insights into the generalizability of trial findings and help inform treatment strategies in varied healthcare settings.

Identifying advanced NSCLC patients with PD-L1 TPS ≥ 50% who benefit most from monotherapy is crucial, as they are also eligible for combination therapies, including chemotherapy, anti-VEGF agents, and dual ICI regimens, which may increase the risk of toxicity [31,32]. Effective patient selection can minimize unnecessary treatment-related adverse effects while maximizing treatment outcomes.

Our findings regarding the association of good performance status (ECOG PS 0–1), high PD-L1 TPS (≥90%), and the occurrence of irAEs with improved survival outcomes are consistent with previously reported studies [12,13,15,26,28,29,37,39]. While these are not entirely novel predictors, our study provides important real-world validation of these associations in a Serbian patient population, which has been underrepresented in the literature. Regional real-world evidence remains crucial to better understand how treatment outcomes may vary across different healthcare systems and patient populations.

## 5. Conclusions

Our real-world multicenter study demonstrated that first-line pembrolizumab monotherapy is effective and well tolerated in patients with metastatic NSCLC and PD-L1 TPS ≥ 50%. The observed median PFS and OS were consistent with, though slightly lower than, those reported in pivotal clinical trials, reflecting the broader patient population encountered in routine clinical practice. Importantly, good performance status (ECOG PS 0–1), very high PD-L1 expression (TPS ≥ 90%), and the occurrence of immune-related adverse events were independently associated with improved survival outcomes. These findings validate known prognostic factors in a real-world Eastern European cohort and may support more personalized treatment approaches for patients receiving pembrolizumab monotherapy.

## 6. Limitations

As a retrospective observational study, certain clinical information was either unavailable or inconsistently recorded in physician charting. Moreover, there are local differences in treatment approaches in included centers, making it more difficult to precisely determine certain variables. Furthermore, for the purpose of analysis, both PD-L1 TPS and age were treated as dichotomous variables, whereas in reality, they are continuous parameters, and this simplification might limit the nuance of our findings. Of note, the patients were only tested for oncogenic alterations in ALK and EGFR genes, as other molecular testing was not part of routine practice in Serbia at the time this study was conducted.

## Figures and Tables

**Table 1:** Demographic data of included patients.

N = 225	N (%)
Mean age at treatment start (range) [years]	64.77 (35–90)
Sex	
Male	144 (64%)
Female	81 (36%)
Smoking status	
Current	133 (59.1%)
Ex smoker	58 (25.8%)
Non-smoker	34 (15.1%)
ECOG PS	
0–1	183 (81.3%)
≥2	42 (18.7%)
Histological diagnosis	
Adenocarcinoma	139 (61.7%)
Squamous cell carcinoma	69 (30.6%)
Other (NOS)	17 (7.7%)
PD-L1 status	
50–89%	196 (87.1%)
90–100%	29 (12.9%)
Corticosteroid use at the time of treatment initiation	
Yes	35 (15.6%)
No	190 (84.4%)
Radiotherapy during treatment	
Yes	60 (26.7%)
No	165 (73.3%)
Patients that experienced immune-related adverse events	
Yes	47 (20.8%)
No	178 (79.1%)
Number of metastatic sites	
Mean	1.4
3<	210 (93.3%)
3≥	15 (6.7%)
CNS metastasis at baseline	
Yes	49 (11.8%)
No	176 (78.2%)

**Table 2:** Comparison of baseline characteristics between group of patients with and without irAEs.

	Patients with irAE (N = 47)	Patients Without irAE (N = 178)	*p* Value
**Sex**			0.97
Male	30 (63.8%)	114 (64%)	
Female	17 (36.2%)	64 (36%)	
**Smoking status**			0.96
Current or former	40 (85.1%)	151 (84.8%)	
Never	7 (14.9%)	40 (15.2%)	
**Age**			0.93
<70 years	33 (70.2%)	126 (70.7%)	
>70 years	14 (29.8%)	52 (29.3%)	
**Histology**			0.10
non-squamous	28 (59.6%)	128 (71.9%)	
squamous	19 (40.4%)	50 (29.1%)	
**Metastatic sites**			0.94
<3	44 (93.6%)	166 (93.2%)	
≥3	3 (6.4%)	12 (6.8)	
**PD-L1 status**			0.37
50–89%	39 (82.9%)	157 (88.2%)	
90–100%	8 (17.1%)	21 (11.8%)	
**ECOG PS**			0.24
0–1	41 (87.2%)	142 (79.7%)	
2	6 (12.8%)	36 (20.3%)	
**Radiotherapy**			0.52
Yes	13 (27.6%)	66 (37%)	
No	34 (72.4%)	166 (63%)	
**Corticosteroid use at the start of therapy**			0.86
Yes	13 (27.6%)	47 (26.4%)	
No	34 (72.4%)	131 (73.6%)	

**Table 3:** Comparison of baseline characteristics between group of patients with ECOG PS 0–1 and ECOG PS ≥ 2.

	Patients with ECOG PS 0–1 (N = 183)	Patients with ECOG PS ≥ 2 (N = 42)	*p* Value
**Sex**			0.69
Male	116 (63.4%)	28 (66.6%)	
Female	67 (36.6%)	14 (33.4%)	
**Smoking status**			0.75
Current or former	156 (85.2%)	35 (83.4%)	
Never	27 (14.8%)	7 (16.6%)	
**Age**			0.01
<70 years	136 (74.3%)	23 (54.7%)	
≥70 years	47 (25.7%)	19 (45.3%)	
**Histology**			0.10
non-squamous	128 (69.9%)	28 (66.6%)	
squamous	55 (30.1%)	14 (33.4%)	
**Metastatic sites**			0.41
<3	172 (93.9%)	38 (90.4%)	
≥3	11 (6.1%)	4 (9.6%)	
**irAE**			0.24
Yes	41 (22.4%)	6 (14.3%)	
No	142 (87.6%)	36 (85.7%)	
**PD-L1 status**			0.21
50–89%	157 (85.7%)	39 (92.8%)	
90–100%	26 (14.3%)	3 (7.2%)	
**Radiotherapy**			0.10
Yes	53 (28.9%)	7 (16.6%)	
No	130 (71.1%)	35 (83.4%)	
**Corticosteroid use at the start of therapy**			0.00
Yes	18 (9.8%)	17 (40.4%)	
No	165 (91.2%)	25 (59.6%)	

**Table 4:** Comparison of baseline characteristics between group of patients with PD-L1 TPS 50–89% and PD-L1 TPS ≥ 90%.

	Patients with PD-L1 TPS ≥ 90% (N = 29)	Patients with PD-L1 TPS 50–89% (N = 196)	*p* Value
**Sex**			0.81
Male	18 (62%)	126 (64.3%)	
Female	11 (38%)	70 (35.7%)	
**Smoking status**			0.75
Current or former	26 (89.6%)	165 (86.8%)	
Never	3 (10.4%)	26 (13.2%)	
**Age**			0.27
<70 years	23 (79.3%)	136 (69.4%)	
≥70 years	6 (20.7%)	60 (30.6%)	
**Histology**			0.21
non-squamous	22 (75.8%)	134 (68.3%)	
squamous	7 (24.2%)	62 (22.7%)	
**Metastatic sites**			0.39
<3	26 (89.6%)	184 (93.8%)	
≥3	3 (10.4%)	12 (6.2%)	
**irAE**			0.34
Yes	8 (27.6%)	39 (19.8%)	
No	21 (72.4%)	157 (81.2%)	
**ECOG PS**			0.24
0–1	26 (89.6%)	157 (80.1%)	
2	3 (10.4%)	39 (19.9%)	
**Radiotherapy**			0.30
Yes	10 (34.5%)	146 (74.5%)	
No	19 (65.5%)	50 (25.5%)	
**Corticosteroid use at the start of therapy**			0.41
Yes	3 (10.4%)	35 (17.9)	
No	26 (89.6%)	161 (82.1)	

**Table 5:** Types and grades of irAEs as per CTCAEv5.

irAE Type (N = 61)	Grade 1–2	Grade 3–4
Colitis	8 (13.1%)	1 (1.6%)
Thyroid toxicities	9 (14.7%)	
Skin toxicity	17 (27.8%)	
Pneumonitis	2 (3.2%)	1 (1.6%)
Hepatotoxicity	4 (6.5%)	4 (6.5%)
Mucositis	7 (11.4%)	
Arthritis	5 (8.1%)	
Myositis	2 (3.2%)	
Polyneuropathy	1 (1.6%)	

**Table 6:** Univariate and multivariate regression analysis of patients for PFS.

	Univariate Regression Analysis			Multivariate Regression Analysis		
	HR	95% CI	*p*	HR	95% CI	*p*
Sex (male vs. female)	0.991	0.734–1.338	0.953			
Age (<70 years vs. ≥70 years)	1.025	0.741–1.418	0.882			
Histology (non-squamous vs. squamous)	1.065	0.774–1.465	0.699			
Smoking status (current or former smoker vs. never-smoker)	0.719	0.488–1.059	0.095	0.785	0.531–1.160	0.225
PD-L1 (50–89% vs. ≥90%)	0.550	0.345–0.876	0.012	0.545	0.338–0.877	0.012
ECOG PS (0–1 vs. ≥2)	4.537	3.140–6.555	0.001	4.409	3.007–6.465	0.001
CNS mets (yes vs. no)	0.979	0.689–1.391	0.907			
Number of metastatic sites (<3 vs. ≥3 localizations)	1.044	0.581–1.875	0.887			
irAE (yes vs. no)	0.638	0.441–0.924	0.017	0.685	0.472–0.997	0.049
Use of corticosteroids at the time of treatment initiation (yes vs. no)	1.595	1.083–2.350	0.018	1.112	0.742–1.666	0.608
Radiotherapy (yes vs. no)	1.090	0.787–1.510	0.602			

**Table 7:** Univariate and multivariate regression analysis of patients for OS.

	Univariate Regression Analysis			Multivariate Regression Analysis		
	HR	95% CI	*p*	HR	95% CI	*p*
Sex (male vs. female)	1.124	0.807–1.567	0.489			
Age (<70 years vs. ≥70 years)	1.355	0.964–1.905	0.080	1.120	0.790–1.586	0.524
Histology (non-squamous vs. squamous)	1.304	0.916–1.854	0.140			
Smoking status (current or former smoker vs. never-smoker)	0.719	0.716–1.178	0.502			
PD-L1 (50–89% vs. ≥90%)	0.511	0.299–0.871	0.014	0.434	0.249–0.757	0.003
ECOG PS (0–1 vs. ≥2)	4.907	3.356–7.174	0.001	5.233	3.505–7.813	0.001
CNS mets (yes vs. no)	1.234	0.848–1.794	0.272			
Number of metastatic sites (<3 vs. ≥3 localizations)	0.829	0.421–1.631	0.587			
irAE (yes vs. no)	0.638	0.429–0.948	0.026	0.602	0.403–0.899	0.013
Use of corticosteroids at the time of treatment initiation (yes vs. no)	1.665	1.089–2.545	0.019	1.141	0.735–1.771	0.557
Radiotherapy (yes vs. no)	1.297	0.911–1.847	0.149			

## Data Availability

The raw data supporting the conclusions of this article will be made available by the authors on request.
